# Suppression of Experimental Choroidal Neovascularization by Curcumin in Mice

**DOI:** 10.1371/journal.pone.0053329

**Published:** 2012-12-28

**Authors:** Ping Xie, WeiWei Zhang, Songtao Yuan, Zhiqiang Chen, Qin Yang, DongQing Yuan, Feng Wang, QingHuai Liu

**Affiliations:** 1 Department of Ophthalmology, The First Affiliated Hospital with Nanjing Medical University, Nanjing, Jiangsu, P.R. China; 2 Department of Ophthalmology, JiangSu Province Official Hospital, Nanjing, Jiangsu, P.R. China; National Eye Institute, United States of America

## Abstract

**Purpose:**

To investigate the effects of curcumin on the development of experimental choroidal neovascularization (CNV) with underlying cellular and molecular mechanisms.

**Methods:**

C57BL/6N mice were pretreated with intraperitoneal injections of curcumin daily for 3 days prior to laser-induced CNV, and the drug treatments were continued until the end of the study. The CNV area was analyzed by fluorescein-labeled dextran angiography of retinal pigment epithelium (RPE)-choroid flat mounts on day 7 and 14, and CNV leakage was evaluated by fluorescein angiography (FA) on day 14 after laser photocoagulation. The infiltration of F4/80 positive macrophages and GR-1 positive granulocytes were evaluated by immunohistochemistry on RPE-choroid flat mounts on day 3. Their expression in RPE-choroid complex was quantified by real-time PCR (F4/80) and Western blotting (GR-1) on day 3. RPE-choroid levels of vascular endothelial growth factor (VEGF), tumor necrosis factor (TNF)-α, monocyte chemotactic protein (MCP)-1, and intercellular adhesion molecule (ICAM)-1 were examined by ELISA on day 3. Double immunostaining of F4/80 and VEGF was performed on cryo-sections of CNV lesions on day 3. The expression of nuclear factor (NF)-κB and hypoxia-inducible factor (HIF)−1α in the RPE-choroid was determined by Western blotting.

**Results:**

Curcumin-treated mice had significantly less CNV area (*P*<0.05) and CNV leakage (*P*<0.001) than vehicle-treated mice. Curcumin treatment led to significant inhibition of F4/80 positive macrophages (*P*<0.05) and GR-1 positive granulocytes infiltration (*P*<0.05). VEGF mainly expressed in F4/80 positive macrophages in laser injury sites, which was suppressed by curcumin treatment (*P*<0.01). Curcumin inhibited the RPE-choroid levels of TNF-α (*P*<0.05), MCP-1 (*P*<0.05) and ICAM-1 (*P*<0.05), and suppressed the activation of NF-κB in nuclear extracts (*P*<0.05) and the activation of HIF−1α (*P*<0.05).

**Conclusion:**

Curcumin treatment led to the suppression of CNV development together with inflammatory and angiogenic processes including NF-κB and HIF−1α activation, the up-regulation of inflammatory and angiogenic cytokines, and infiltrating macrophages and granulocytes. This provides molecular and cellular evidence of the validity of curcumin supplementation as a therapeutic strategy for the suppression of age-related macular degeneration (AMD)-associated CNV.

## Introduction

Age-related macular degeneration (AMD) is the leading cause of blindness among elderly people in developed countries [1]. Most of the severe vision loss that can be attributed to AMD results from its exudative form, which is characterized by choroidal neovascularization (CNV). CNV is defined as the penetration of immature new blood vessels into the Bruch’s membrane from choriocapillaries and their extension into the sub-retinal and/or sub-retinal pigment epithelium (RPE) space. This is associated with manifestations such as RPE detachment, subretinal hemorrhages, and fibrovascular disciform scarring [2].

Although the pathogenic mechanisms underlying CNV are complex and still largely unknown, increasing amounts of evidence indicate that inflammatory and angiogenic events, including inflammatory cells infiltration and cytokine networks, play crucial roles in the development of CNV [3]–[5]. Experimental and clinical studies have revealed that macrophages accumulate in CNV area and express a variety of cytokines, including vascular endothelial growth factor (VEGF), which is recognized as a key signal in promoting angiogenesis and critical for CNV formation [6]–[8]. In mice that show pharmacologic depletion of macrophages, CNV is reduced when VEGF is reduced [3]–[9]. Granulocytes also have been found to influx into laser induced CNV lesions and its depletion correlated with reduced CNV responses and decreased VEGF protein expression [10]–[11]. These data suggests an important role for macrophages and granulocytes in the pathogenesis of CNV. CNV tissues from human surgical extracted samples and the rodent laser-induced models also express high levels of numerous inflammation and angiogenesis-related cytokines, such as monocyte chemoattractant protein-1 (MCP-1; also known as a major chemokine for macrophages), intercellular adhesion molecule (ICAM)-1, and tumor necrosis factor (TNF)-α [6], [12]–[18]. Neutralization of TNF-α by its monoclonal antibody or genetic ablation of MCP-1 or ICAM-1, the size and leakage of laser-induced CNV were significantly reduced [16], [19]–[21]. Moreover, some inflammation-related or angiogenesis-related transcription factors, such as nuclear factor (NF)-κB and hypoxia-inducible factor (HIF)−1α, are reported to involve in the pathogenesis of CNV, and their inhibition result in the reduction of laser-induced CNV [22]–[23]. All of these lines of evidence suggest the regulation of inflammation and angiogenesis as the important therapeutic strategy in the suppression of CNV.

Curcumin is a low-molecular-weight phenolic compound originating from turmeric (*Curcuma longa*). It has been used for centuries as a wound-healing agent and for treating various illnesses in traditional Indian and Chinese medicine [24]. In recent years, extensive *in vitro* and *in vivo* studies have demonstrated that curcumin possesses a wide variety of biological activities, including anti-cancer, anti-oxidant, anti-inflammatory and anti-angiogenic properties [25]–[27]. The underlying mechanisms of these effects are diverse and involve the regulation of various inflammation-related molecular targets and cellular targets, including those mentioned above, which can be crucial to the pathogenesis of CNV. Due to its efficacy, ability to affect multiple targets and to its known safety for human use, curcumin has showed the potent therapeutic value in clinical settings in the prevention and treatment of various chronic and acute inflammatory diseases, such as rheumatoid arthritis, psoriasis, inflammatory bowel disease, and acute rejection in kidney transplantation [28]–[32]. Moreover, in the treatment of inflammatory eye diseases in humans, curcumin has been shown to be as effective as corticosteroids for chronic anterior uveitis [33], to be effective in the management of chronic anterior uveitis relapses [34], and to reduce or resolve inflammatory orbital pseudotumors [35].

Considering the crucial roles of inflammation in neovascularization, we hypothesized that curcumin might represent a potential agent for the treatment of CNV. In this study, we administered curcumin to a laser-induced mouse model of CNV to determine whether this compound can prevent the formation of CNV formation. We also investigated possible underlying cellular and molecular mechanisms.

## Materials and Methods

### Animals

Male wild-type C57BL/6 mice (Nanjing Medical University Laboratory Animal Center, China) 8 weeks of age were used as the laser induced CNV mouse model. The mice were anesthetized with sodium pentobarbital, and the pupils were dilated with topical 1% tropicamide (Santen, Osaka, Japan). This study was carried out in strict accordance with the recommendations in the guide for the care and use of animals of the Association for Research in Vision and Ophthalmology (ARVO). The protocol was approved by the Committee on the Ethics of Animal Experiment of the First Affiliated Hospital of Nanjing Medical University (Permit Number: 22-005029). All reasonable efforts were made to minimize suffering.

### Drug Treatment and Laser-induced CNV

Mice were pretreated with curcumin (C1386, Sigma-Aldrich) or vehicle (dimethyl sulfoxide dissolved in phosphate buffered saline, 0.1%) for 3 days before photocoagulation and the treatment was continued until the end of the study. Firstly, curcumin was administered to mice by peritoneal injection with the dose of 10, 30 or 90 mg/kg body weight per day to evaluate the inhibitory effect of curcumin on CNV size. Based on this result, 30 mg/kg curcumin was determined in other experiments.

Laser photocoagulation (532 nm Argon laser, 120 mW, 100 ms duration, 75 µm spot size; Novus® Varia™, UT, U.S.) was performed bilaterally in each mouse. Laser spots were applied in a standard fashion around the optic nerve using a slit lamp delivery system (Lumenis 1000, UT, U.S.) using a handheld cover slip as a contact lens. Only burns that produced a bubble, indicating the rupture of the Bruch’s membrane, were included in the study.

### Quantification of Laser-induced CNV

On day 7 and day 14 after laser photocoagulation, the sizes of CNV lesions were measured on RPE-choroid flat mounts by fluorescein-labeled dextran (*#*FD2000S-1G, Sigma, MO, U.S.) perfusion. In brief, mice were deeply anesthetized and perfused via left ventricle with 1 ml PBS containing 50 mg/ml fluorescein-labeled dextran. Then mice were killed, and the eyes were enucleated and fixed in 4% paraformaldehyde (PFA) for 1 hour. After washing in PBS, the anterior segment of the eye was cut off, and the whole retinas were carefully removed from the eyecups. Four radial cuts in the remaining RPE-choroid-sclera were made from the edge to the equator, and the eyecups were flat-mounted with the RPE layer facing up. Those flat mounts were examined and recorded using the same microscope as earlier. An examination was performed of the 66 spots from 12 vehicle treated mice and of 60 spots from 12 curcumin-treated mice were examined, excluding eyes with hemorrhages (2 eyes in vehicle treated mice and 4 eyes in curcumin-treated mice). Image J for Windows (NIH, Bethesda, MD, U.S.) analysis software was used to measure the area of CNV. Operators were blinded with respect to treatment groups.

### Fundus Photography and Fluorescein Angiography (FA)

To confirm the inhibitory effect of 30 mg/kg curcumin on CNV formation, fluorescein angiography was performed on day 14 after laser photocoagulation. Fundus examinations were performed under systemic anesthesia and pupil dilation using a digital fundus camera (Heidelberg Retina Angiograph II, HRA 2, CA, U.S.), and the laser lesions were studied using fluorescein angiography to evaluate CNV development and its activity. The fluorescein sodium injection (10%; 0.1 ml/kg; Alcon, TX, U.S.) was injected into the intraperitoneal cavity of the mice, and fundus angiogram photographs were recorded using a High Performance Digital Image System VK-2 (Kowa, Japan). Fluorescein leakage was defined as the presence of a hyper-fluorescent lesion that increased in size over time in the late-phase angiogram as previously described [36]. Angiograms were graded as follows: 0-no leakage, 1-slight leakage, 2-moderate leakage, 3-prominent leakage. The number of leaky lesions was counted and the extent of fluorescein leakage was graded in a masked fashion by two examiners.

### Immunohistochemistry of Macrophages and Granulocytes on RPE-choroid Flat Mounts

To identify possible cellular responses to curcumin administration in the CNV model, we examined macrophages and granulocytes accumulation in laser injury sites. Macrophages were detected using F4/80 antibody and granulocytes were detected using GR-1 antibody in the RPE -choroid flat mounts on day 3. In brief, 2 mice per group were killed on day 3 after laser photocoagulation, and the eye cups were prepared as given above. The eyecups were fixed with 4% PFA for 8 hours and dehydrated in 30% sucrose for 6 hours at 4°C. After blocking with 5% BSA for 1 hour, these eye cups were incubated with primary antibodies against mouse F4/80 (1∶500, Catalog No. BM40075, Monoclonal Antibody to Mouse Macrophages: F4/80, Acris, Germany) or GR-1 (1∶200, Catalog No. MA1-70099, Monoclonal Antibody to Mouse Granulocytes, Thermo Scientific, U.S.) for 24 hours. After washing, the eyecups were respectively incubated with Alexa 546 or Alexa 488–tagged secondary antibodies (Invitrogen, Carlsbad, CA, U.S.) overnight. The eyecups were washed again and mounted as before. These slides were examined under a fluorescence microscope (BX41; Olympus, Tokyo, Japan).

### Quantification of Macrophages by Real-time PCR Analysis (qPCR) and Granulocytes by Western Blot

To quantify macrophages levels, total RNA was extracted from RPE-choroid complexes of 5 mice in each group on day 3 according to the manufacturer’s recommendations (RNeasy Mini-Kit, Cat. No. 74104 Qiagen, Valencia, CA, U.S.). The concentration of RNA was determined by ND-2000 (Nano Drop, U.S.). Total RNA was reverse-transcribed into cDNA according to the manufacturer’s instructions (SuperScript™ III First-Strand Synthesis SuperMix for qRT-PCR, Cat. No. 11752-050, Invitrogen, Carlsbad, CA, U.S.). Real-time PCR was performed using a real-time PCR cycler (ABI Prism 7800 Sequence Detection System; Applied Biosystems, Foster City, CA, U.S.). Relative F4/80 (Mm 00802529 _m1) mRNA levels are calculated for fold induction of gene expression in treated mice in comparison with untreated normal mice, after normalization to GAPDH gene (Mm99999915_g1) using the ΔΔ CT methods described by the manufacturer (Applied Biosystems, Foster City, CA, U.S.).

To quantify granulocytes levels, the RPE-choroid complexes of 5 mice were micro-surgically isolated on day 3 and placed immediately into 200 µl RIPA buffer (R0278, Sigma) supplemented with 1% protease inhibitor cocktail (P8340, Sigma) at 4°C. After mechanical disruption, lysates were placed on ice for 20 minutes and centrifuged at 12,000 rpm for 10 minutes at 4°C. The supernatants were collected and preserved at −70°C. Protein concentrations were determined using a Coomassie Bradford Protein Assay Kit (Catalog No. 23200, Pierce, U.S.). Then 15 µg of total protein per sample was diluted with Laemmli Sample Buffer (Catalog No.161-0737, Bio-Rad, CA, U.S.), heated at 95°C for 5 min, separated by SDS-PAGE (sodium dodecyl sulfate polyacrylamide gel electrophoresis) and electroblotted onto polyvinylidene fluoride membrane (PVDF, GE Healthcare, Buckinghamshire, U.K.). After blocking with 2.5% skim milk for 1 hour at room temperature, the membranes were incubated with the same GR-1 antibody (1∶500) as above or β-actin antibody (1∶1000, Catalog No.4967L, Cell Signaling) overnight at 4°C. After washing with 0.1% Tris-buffered saline (TBS)-Tween, blots were incubated with horseradish peroxidase (HRP)-conjugated goat anti-rat IgG (1∶1000, Catalog No. 7077, Cell Signaling) or goat anti-rabbit IgG (1∶2500, Catalog No. 7074, Cell Signaling) for 1 h at room temperature. The blots were then washed three times with 0.1% TBS-Tween and the signals were visualized using an ECL kit (GE Healthcare, Buckinghamshire, U.K.) according to the manufacturer’s protocol. The densities of immunoreactive bands were measured using Image J for Windows (NIH, Bethesda, MD, U.S.).

### Enzyme-linked Immunosorbent Assay (ELISA) of VEGF, TNF-α, MCP-1, and ICAM-1

To quantify VEGF, TNF*-*α, MCP-1, and ICAM-1 protein levels, we extracted protein from the RPE-choroid complexes on day 3. The protein extraction and the concentration calculation were the same as the above protocols. The VEGF, TNF*-*α, MCP-1, and ICAM-1 protein levels in the supernatant were determined using mouse VEGF, TNF*-*α, MCP-1, and ICAM-1 ELISA kits (Quantikine; R&D Systems) at 450 nm to 570 nm with an absorption spectrophotometer (BIO-RAD microplate reader 680, U.K.). They were and normalized to total protein, according to the manufacturer’s protocols. Four eyes were needed to extract one protein sample, and 12 mice in each group were examined.

### Immunohistochemistry of Macrophages and VEGF on Cryo-sections

To determine the role of macrophages in VEGF production, double immunostaining of macrophages and VEGF were performed using F4/80 antibody and VEGF antibody on cryo-sections on day 3. In brief, 2 mice per group were killed on day 3 after laser photocoagulation, and the eyes were fixed with 4% paraformaldehyde (PFA) for 8 hours and dehydrated in 30% sucrose for 6 hours at 4°C and then embedded in surgipath FSC 22 ® frozen section medium (Catalog No.3801480, Leica). The eyecups were sectioned into slices with the thickness of 8 µm. After blocking with 5% BSA for 1 hour, these cryo-sections were incubated with the primary antibodies of F4/80 (the same as above) and VEGF (1∶200, Catalog No. ab46154, Rabbit polyclonal to mouse VEGF, Abcam, USA) for 24 hours. After washing, the cryo-sections were incubated with Alexa 488- and 546–tagged secondary antibodies (Invitrogen, Carlsbad, CA, U.S.) and DAPI for 1 hour. These slides were washed again and mounted as before, and examined under the fluorescence microscope as above.

### Western Blot Analysis of NF-κB and HIF−1α

To determine whether curcumin treatment affected the NF-κB signaling pathway in the laser-induced CNV model, the nuclear extract of RPE-choroid complexes from 5 mice were prepared for western blot analysis 6 hours after laser photocoagulation, which was performed according to the method of Andrews [37]. Protein preparation for HIF−1α and the protocols of western blot were the same as the above, except the first antibody and the second antibody. Here, the membranes were incubated with the first antibody of a rabbit polyclonal anti-NF-κB p65 antibody (1∶1000, Catalog SC-109, Santa Cruz, CA, U.S.) or a rabbit monoclonal anti-HIF−1α antibody (1∶500, Catalog 100–105, Novus, U.S.) or a anti-GAPDH (14C10) (1∶1000, Catalog No.2118, Cell Signaling), and the second antibody of a horseradish peroxidase (HRP)-conjugated goat anti-rabbit IgG (1∶2500, Catalog No. 7074, Cell Signaling).

### Statistical Analysis

Results are expressed as the mean ± SE with *n* as indicated. Student’s *t* test and one*-*way ANOVA were used for statistical comparisons of two groups and multiple groups. Differences between the means were considered statistically significant at *P<*0.05.

## Results

### Suppression of CNV Formation in Mice Treated with Curcumin

Curcumin treatment did not induce any significant side effects in present study, such as weight loss, severe infection and death. However, clumps of curcumin precipitations were detected in abdominal cavity of all the mice treated with 90 mg/kg curcumin on day 7 and day 14, while in 10 mg/kg or 30 mg/kg curcumin treated mice, no curcumin precipitations were detected (Data not shown).

**Figure 1:**
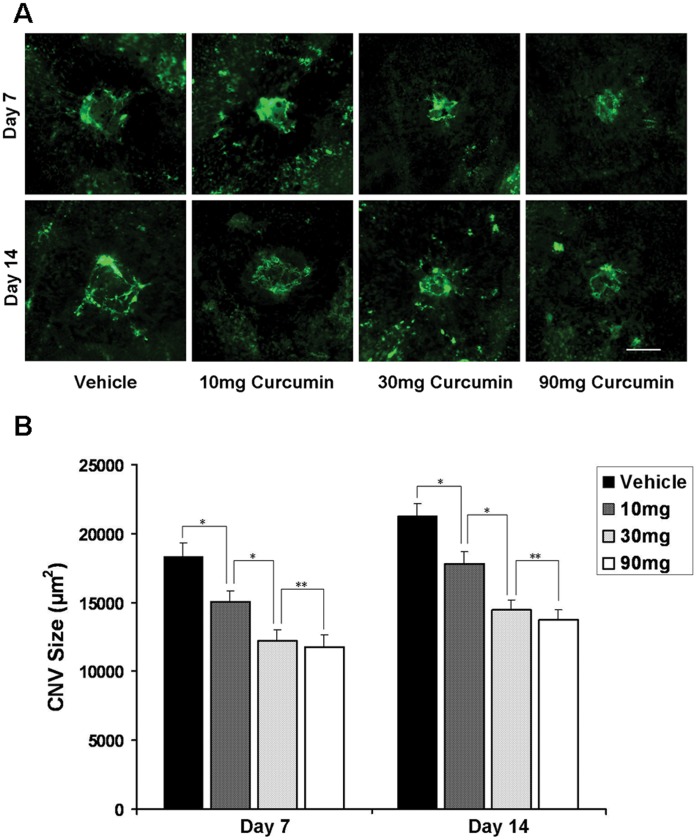
Effects of curcumin on CNV formation. (**A**) Representative micrographs of CNV lesions in the RPE–choroid flat mounts from vehicle-treated mice and curcumin-treated mice on day 7 and day 14 after laser photocoagulation. CNV is indicated by green fluorescence and FITC-dextran angiography. Scale bar = 100 µm. (**B**) Quantitative analysis of CNV size in RPE–choroid flat mounts. Day 7: Vehicle, *n* = 42 spots; 10 mg/kg curcumine, *n* = 45 spots; 30 mg/kg curcumine, *n* = 42 spots; 90 mg/kg curcumine, *n* = 39 spots. Day 14: Vehicle, *n* = 66 spots; 10 mg/kg curcumine, *n* = 54 spots; 30 mg/kg curcumine, *n* = 60 spots; 90 mg/kg curcumine, *n* = 63 spots. *P<0.05; **P>0.05.

An analysis of RPE-choroid flatmounts showed the distinct reduction in CNV area after curcumin treatment (Fig. 1A). On day 7, the mean CNV area was 18,317.10±1014.13 µm^2^ in vehicle-treated mice (*n* = 42 spots). It significantly decreased in 10 mg/kg curcumin-treated mice (15,019.48±823.40 µm^2^, *n* = 45 spots), 30 mg/kg curcumin-treated mice (12,186.47±836.01 µm^2^, *n* = 42 spots) and 90 mg/kg curcumin-treated mice (11,745.97±883.89 µm^2^, *n* = 39 spots). They respectively translated into 18%, 33% and 36% decrease in CNV area by curcumin treatment (*P*<0.05) (Fig. 1B). On day 14, the mean CNV area was 21,300.53±894.57 µm^2^ in vehicle-treated mice (*n* = 66 spots). It also significantly decreased in 10 mg/kg curcumin-treated mice (17,821.71±819.62 µm^2^, *n* = 54 spots), 30 mg/kg curcumin-treated mice (14,445.55±709.74 µm^2^, *n* = 60 spots) and 90 mg/kg curcumin-treated mice (13,752.26±680.79 µm^2^, *n* = 63 spots). They translated into 16%, 32% and 35% decrease in CNV area by curcumin treatment (*P*<0.05) (Fig. 1B).

Compared with the dose of 10 mg/kg, curcumin at the dose of 30 mg/kg and 90 mg/kg showed higher inhibitory effect (P<0.05) both on day 7 and day 14. Although CNV size of 90 mg/kg curcumin treated mice tend to be smaller than that of 30 mg/kg curcumin treated mice, they had no significant difference in statistics (P>0.05).

### Reduction of CNV Leakage Under Curcumin Treatment

**Figure 2:**
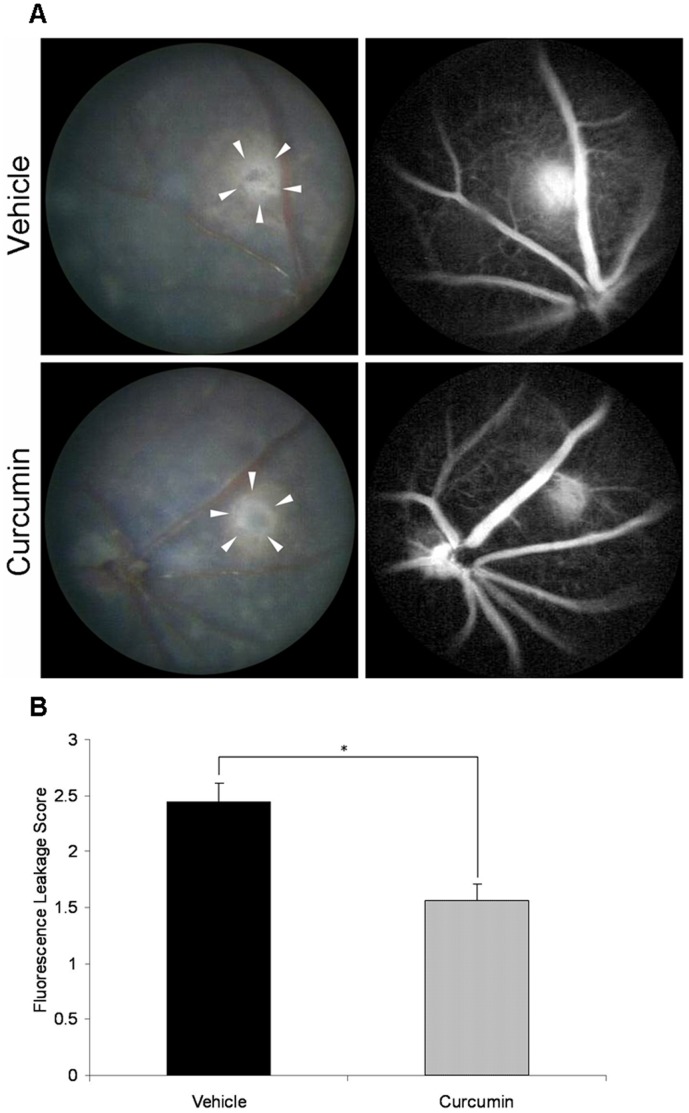
Fluorescein angiography (FA) of CNV lesions. (A) Representative images of fundus and late-phase FA of vehicle-treated and curcumin-treated mice on day 14 after laser photocoagulation (white arrowheads: laser spots). (B) Comparison of semi-quantitative CNV FA score between vehicle- and curcumin-treated mice (*n* = 18 spots, *P<0.001).

All laser spots, which showed a central depigmented crater surrounded by irregular hyperpigmentation in fundus photograph, demonstrated evident fluorescein leakage in FA on day 14 after photocoagulation. Comparisons of fluorescein angiograms between vehicle- and curcumin-treated mice confirmed that the formation of CNV was less severe in curcumin-treated mice (Fig. 2A). Curcumin treatment significantly reduced fluorescein leakage from the photocoagulated lesions by approximately 36% (*n* = 18 spots, *P*<0.001, Fig. 2B).

### Inhibition of Macrophages and Granulocytes Infiltration Under Curcumin Treatment

**Figure 3:**
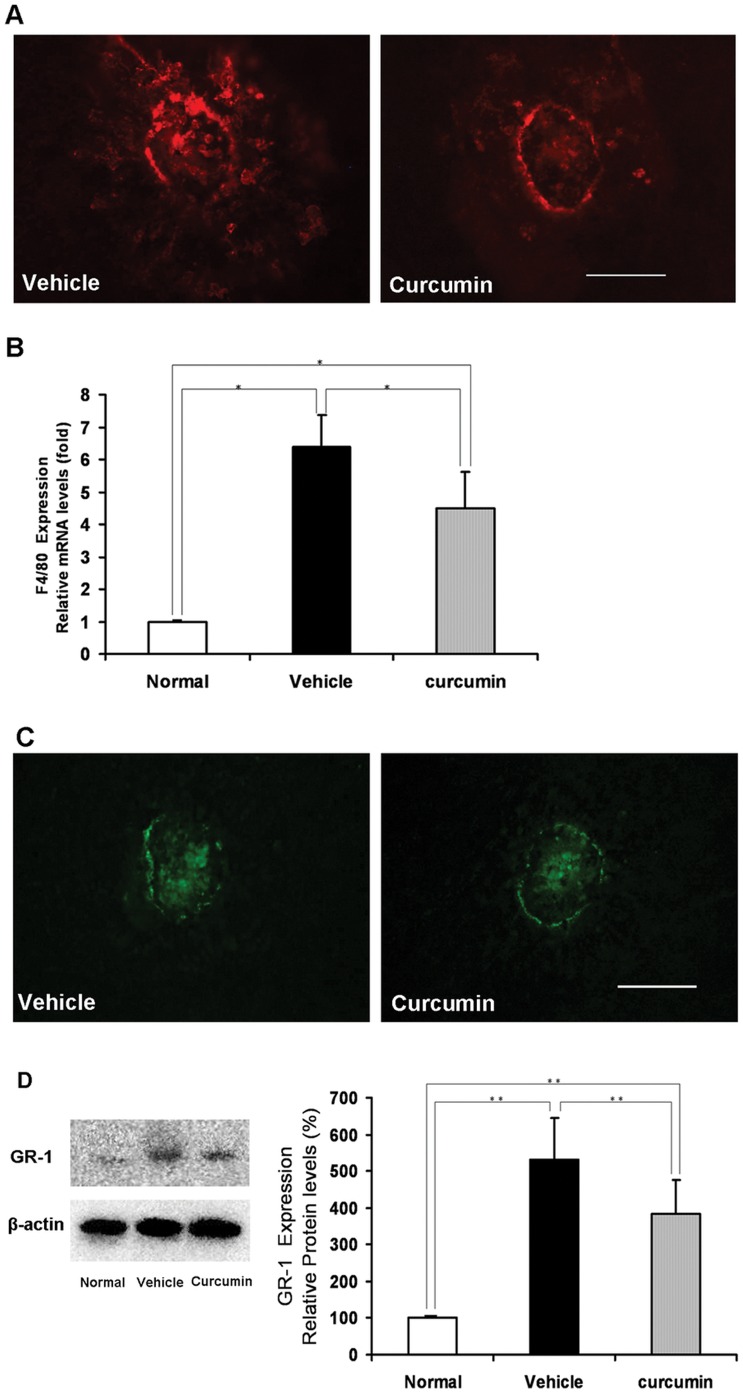
Inhibitory effect of curcumin on macrophages and granulocytes infiltration into CNV. (A) Immunohistochemistry of macrophages (F4/80, red) in RPE–choroid flat mounts on day 3 (Scale bar = 100 µm). (B) The expression of F4/80 mRNA in RPE–choroid complexes on day 3 after photocoagulation. After photocoagulation, F4/80 mRNA expression significantly increased compared with no laser photocoagulation controls (relative to normal control). The increased F4/80 mRNA expression was significantly suppressed by curcumin treatment (*n* = 5, *P<0.05). (C) Immunohistochemistry of granulocytes (GR-1, green) in RPE–choroid flat mounts on day 3 (Scale bar = 100 µm). (D) Left: Representative Western blot showing GR-1 protein expression in samples from vehicle- and curcumin-treated mice on day 3 after photocoagulation. β-actin was used as a loading control. Right: Semi-quantitative analysis of the intensities of GR-1 bands from vehicle- and curcumin-treated mice. The mean for GR-1 in RPE–choroid complex of untreated mice was set at 100% (*n* = 5, **P<0.05).

In the immunohistochemistry of RPE-choroid flatmounts, F4/80-positive macrophages (Fig. 3A) and GR-1-positive granulocytes (Fig. 3C) were substantially rarer in curcumin-treated mice than in vehicle-treated mice.

In quantitative analyses, we detected very low-level F4/80 mRNA expression and GR-1 protein expression in RPE-choroid complexes in normal mice (without laser treatment). On day 3 after laser photocoagulation, the expression of F4/80 and GR-1 dramatically increased, but curcumin treatment significantly suppressed their expression over vehicle treatment (*n* = 5, *P*<0.05, respectively, Fig. 3B and Fig. 3D).

### Inhibition of Angiogenic and Inflammatory Molecules by Curcumin Treatment

**Figure 4:**
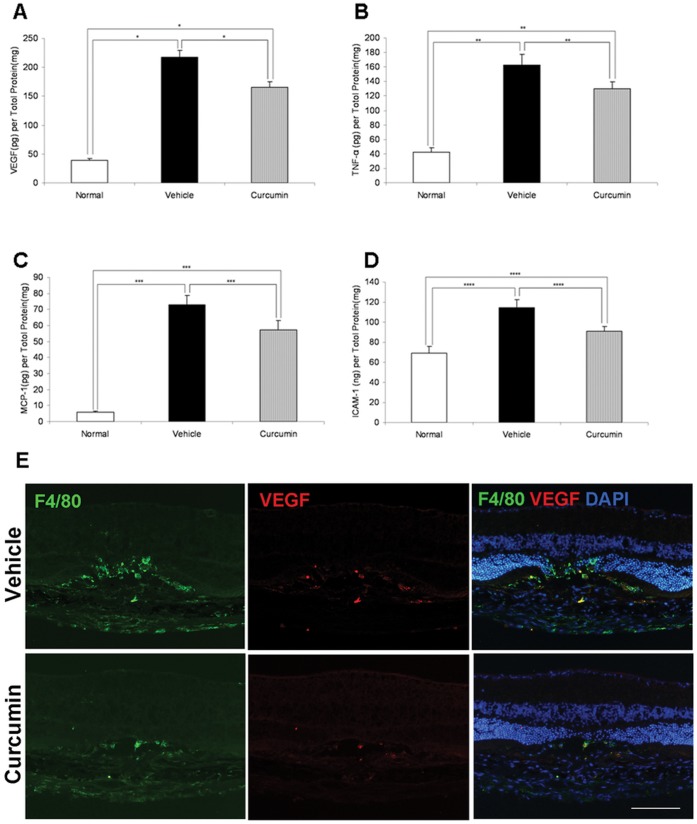
Inhibitory effects of curcumin on RPE–choroid production of angiogenic and inflammatory molecules. Curcumine significantly suppressed RPE–choroid protein levels of VEGF (**A:** *n* = 6. *P<0.01), TNF*-*α (**B:** *n* = 6. **P<0.05), MCP-1 (**C:** *n* = 6. ***P<0.05), and ICAM-1 (**D:** *n* = 6. ****P<0.05) on day 3. (E) Double immunostaining of F4/80 and VEGF on cryo-sections on day 3. High levels of VEGF were expressed in F4/80-positive macrophages at the photocoagulated sites. VEGF localized mainly in infiltrating macrophages at the laser injury sites. Curcumin treatment apparently decreased VEGF immunoreactivity compared to vehicle treatment. Scale bar = 100 µm.

To determine whether curcumin treatment affects angiogenic and inflammatory molecules related to the pathogenesis of CNV, protein levels of VEGF, TNF-α, MCP-1, and ICAM-1 in the RPE–choroid complex were analyzed by ELISA. RPE–choroid levels of VEGF, TNF*-*α, MCP-1, and ICAM-1 were significantly higher in mice with CNV than in age-matched healthy controls. Curcumin treatment significantly suppressed protein levels of VEGF (217.83±11.79 pg/mg *vs.* 165.83±9.16pg/mg, *P*<0.01, *n* = 6, Fig. 4A), TNF*-*α (162.67±14.54 pg/mg *vs.* 129.67±9.98 pg/mg, *P*<0.05, *n* = 6, Fig. 4B), MCP-1 (72.83±6.04pg/mg *vs.* 57.17±6.02 pg/mg, *P*<0.05, *n* = 6, Fig. 4C), and ICAM-1 (114.50±7.91 pg/mg *vs.* 90.67±5.08 pg/mg, *P*<0.05, *n* = 6, Fig. 4D) relative to vehicle treatment.

In immunohistochemistry, strong VEGF-positive immunoreactivity was detected in the laser injury sites. The immunoreactivity was mainly localized to infiltrating F4/80-positive macrophages at the laser injury site. Curcumin treatment decreased the VEGF immunostaining with the reduction of F4/80-positive macrophages compared to vehicle treatment (Fig. 4E).

### Suppression of NF-κB and HIF−1α by Curcumin Treatment

**Figure 5:**
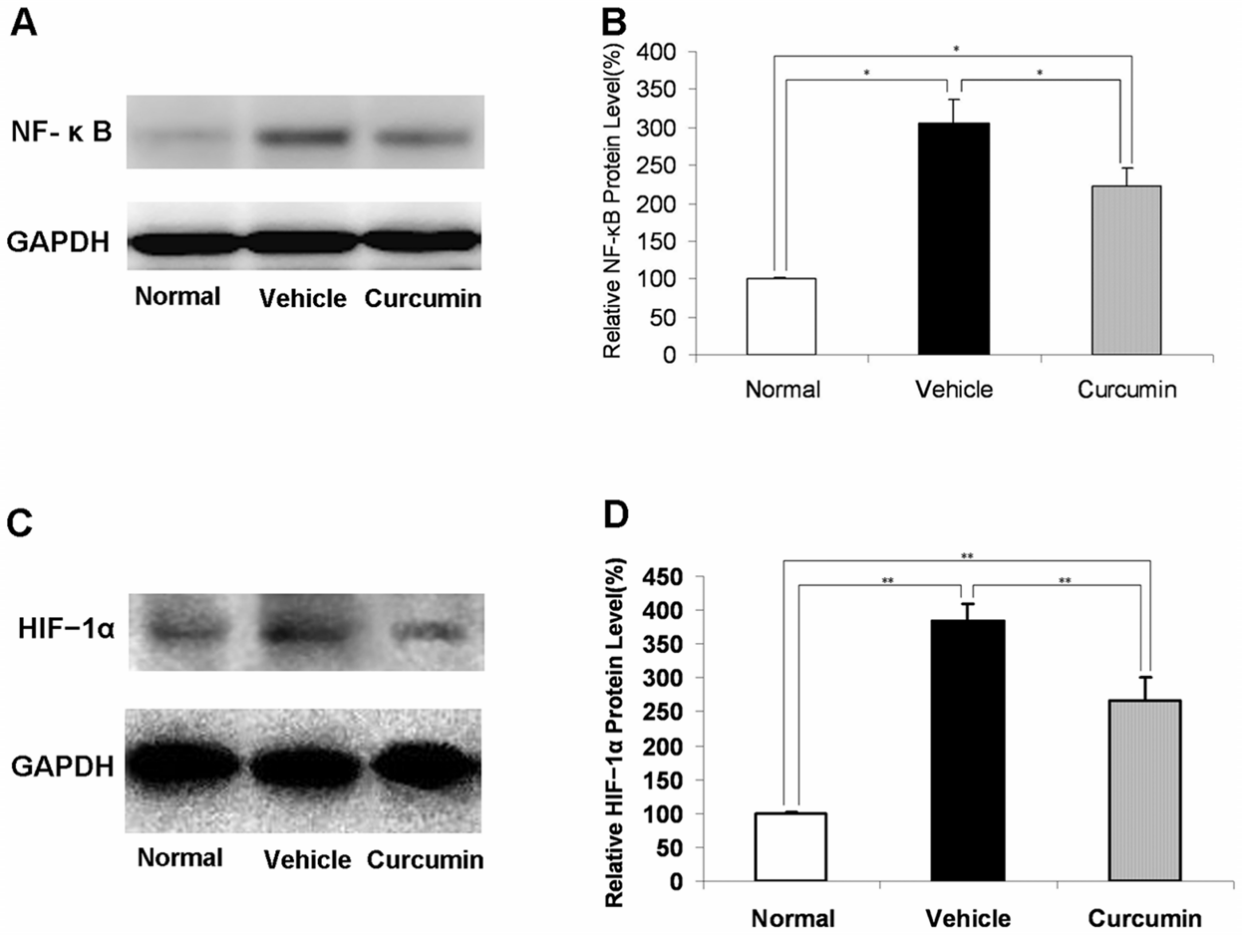
Suppression of NF-κB and HIF−1α by curcumin treatment. (A) Representative Western blot showing NF- κB protein in the nuclear extract in samples from vehicle- and curcumin-treated mice 6 hours after laser injury. GAPDH was used as a loading control. (B) Semiquantitative analysis of the intensities of NF- κ B bands in nuclear extracts from vehicle- and curcumin-treated mice. The mean for NF- κ B in RPE–choroid complex of untreated mice was set at 100% (*n* = 5, *P<0.05). (C) Representative Western blot showing HIF−1α protein expression in samples from vehicle- and curcumin-treated mice on day 3 after photocoagulation. GAPDH was used as a loading control. (D) Semiquantitative analysis of the intensities of HIF−1α bands from vehicle- and curcumin-treated mice. The mean for HIF−1α in RPE–choroid complex of untreated mice was set at 100% (*n* = 5, **P<0.05).

To investigate the signaling pathway involved in curcumin treatment, we focused on NF-κB as an upstream transcriptional factor of inflammatory mediators and analyzed nuclear translocation of NF-κB p65. In nuclear extracts from mice with no laser injuries, NF-κB expression was very low. However, it had increased significantly 6 hours after laser photocoagulation, and this increase was reduced by curcumin treatment (*n* = 5, *P*<0.05, Fig. 5A, B). As an transcriptional factor of angiogenic mediators, HIF−1α protein expression significantly increased in choroid-RPE complexes on day 3 after photocoagulation, and it was markedly suppressed by curcumin treatment (*n* = 5, *P*<0.05, Fig. 5C, D).

## Discussion

In the present study, we evaluated the therapeutic value of curcumin in the treatment of a mouse model of laser-induced CNV. Our results demonstrated that curcumin-treated mice had smaller CNV size and less fluorescence leakage than the vehicle-treated mice, which suggest that curcumin can effectively suppress the experimental CNV. Dose response to curcumin treatment showed that the dose of 30 or 90 mg/kg has higher inhibitory effect on CNV size than that of 10 mg/kg, however, no significant difference were detected between the former 2 doses. This maybe caused by the precipitation of curcumin at the dose of 90 mg/kg, which decrease the bioavailability of curcumin. At this dose (90 mg/kg), it is also difficult to gauge how much of the injected curcumin had been absorbed. Therefore, we determined the dose of 30 mg/kg to investigate the possible underlying cellular and molecular mechanisms.

To investigate the possible cellular mechanism underlying curcumin suppression in CNV, we evaluated the infiltration of macrophages and granulocytes on the day of peak response (Day 3) [10], [12]–[38]. Results demonstrated that the infiltration of macrophages and granulocytes was significantly suppressed by curcumin treatment. During the early phase of laser-induced CNV, local MCP-1 was found to increase and recruit monocytes to the site of laser injury sites [39]–[40]. There they become inflammatory macrophages expressing various angiogenic cytokines and inflammatory cytokines, and promote neovascularization [22]–[41]. Granulocytes are another infiltrating cells in laser lesions, which act as the potent initiator of inflammation and angiogenesis in the early phase of laser-induced CNV [10]–[11].The depletion of macrophages or granulocytes can result in significant suppression of CNV formation [3], [9]–[10]. As a potent anti-inflammatory agent, curcumin has displayed the ability to inhibit macrophages migration *in vitro*, and suppress macrophage infiltration in a variety of preclinical animal models of inflammation-associated diseases, such as diabetic or obstructive nephropathy, lipopolysaccharide- or high-glucose-induced renal inflammation and obesity-induced inflammation [42]–[46]. Curcumin also has been reported to block the chemotaxis of granulocytes *in vitro* [47], and inhibit influx of granulocytes in a series of chronic and acute inflammatory diseases in animal model, such as inflammatory bowel disease [48], pancreatitis [49], airway inflammation and lung cancer [50], arthritis [51] and shock [52]. Our results are consistent with these data and indicate that the suppression of macrophages and granulocytes infiltration acts as an important cellular mechanism for curcumin treatment in the current model.

A variety of cytokines, chemokines, and endothelial adhesion molecules, along with cellular behavior, orchestrate the formation of CNV [53]. In the present study, we investigated the impact of curcumin on the production levels of the angiogenesis- and inflammation-associated molecules underlying macrophages, including VEGF, TNF-α, MCP-1, and ICAM-1, which has been shown to be up-regulated in both human and animal CNV tissues. VEGF is a potent angiogenic stimulator that promotes proliferation and migration of vascular endothelial cells and enhances vascular permeability [54]. Previous reports regarding the molecular mechanisms underlying the development of CNV showed VEGF to be a crucial promoting mediator [5]–[8]. Curcumin has shown to inhibit VEGF production in numerous inflammation-associated animal models of disease, such as diabetic retinopathy, corneal neovascularization, diabetic nephropathy, and ectopic endometrium [27] [55]–[59]. Consistent with these data, our results demonstrate that curcumin can decrease VEGF production in the early phase of laser-induced CNV. In addition, it has been reported that the infiltrating macrophages in CNV lesions are a rich source of VEGF and that curcumin can suppress VEGF production in stimulated monocyte cells *in vitro* [4]–[60]. Our results of VEGF and macrophages double immunostaining agree with these data and indicate that macrophages play an important role in the variation of intro-ocular VEGF after laser injury. Therefore, in this study, the curcumin-induced suppression of the expression of VEGF after laser injury can be explained at least in part by treatment with curcumin, effectively inhibiting the infiltration of macrophages secreting VEGF.

Our results also show that curcumin significantly inhibited the protein levels of TNF-α, MCP-1, and ICAM-1 in the RPE-choroid complexes with CNV. TNF-α expressed in infiltrating macrophages has been found in surgically excised CNV membranes of patients with AMD [18]–[61]. The size and leakage of laser-induced CNV lesions were reduced by TNF-α inhibitors in mice [21], rats [62], and monkeys [63]. Moreover, anti-TNF-α therapy in patients with inflammatory arthritis who also had AMD resulted in partial CNV regression and improvements in visual acuity [64]–[65]. These studies have highlighted the role of TNF-α in CNV pathogenesis. TNF-α is a pleiotropic cytokine that mediates angiogenic and inflammatory effects in the cells involved in the formation of CNV. For example, TNF-α induces VEGF production from monocytes and RPE cells [60]–[61]. In addition, TNF-α can increase the production of inflammatory cytokines from RPE cells and endothelial cells, including MCP-1 and ICAM-1 [66]–[70]. MCP-1 is one of the most potent macrophages recruiting molecules, and ICAM-1 is an important component of cell-to-cell interactions during inflammatory responses, mediating leukocyte (including macrophages) adhesion [41]–[71]. Both MCP-1 and ICAM-1 have been shown to be associated with the progression of CNV [6]–[67], [72]–[73]. Previous studies have shown that curcumin can inhibit the production of TNF-α in lipopolysaccharide (LPS)- or phorbol methyl acetate (PMA)-stimulated dendritic cells, monocytes, macrophages, endothelial cells, and bone marrow cells and inhibited MCP-1 and ICAM-1 expression in TNF-α stimulated endothelial cells [66]–[74]. In diabetic rats, curcumin significantly reduced TNF-α levels in the retina, prevented experimental diabetic retinopathy, decreased MCP-1 and ICAM-1 levels in the kidney, and ameliorated macrophage infiltration [43]–[56]. Our results are consistent with these *in vitro* and *in vivo* studies and confirm the profound inhibitory effects of curcumin on these inflammatory cytokines.

Because transcription factor NF-κB is known to regulate the expression of a wide range of genes critical to inflammation, we examined the effects of curcumin on the NF-κB expression in nuclear extracts of RPE-choroid complexes after photocoagulation [75]. Previous studies have shown that NF-κB becomes activated with the up-regulation of angiogenic or inflammatory molecules, such as VEGF, MCP-1, ICAM-1, and IL-6, during the early phase of CNV formation [22]–[76]. NF-κB inhibition leads to significant suppression of experimental CNV [22]. Curcumin has been shown to prevent NF-κB activation in numerous types of cells, including macrophages, RPE cells, and endothelial cells, and then suppresses various inflammation-associated gene products [77]–[80]. Our results show that curcumin suppress the activation of NF-κB in the RPE-choroid complexes with CNV, which is consistent with the results of previous reports. HIF-1α, the main reactor of hypoxia, can activate the transcription of various angiogenic genes including VEGF and ICAM-1, and plays a pivotal role in angiogenesis [81]. HIF-1α expression was detected in surgically excised human CNV membranes [82]–[83] and its level elevated in the eyes of laser induced CNV animal models (23, 18032914, 22915031) [23], [81]–[84]. While in pharmacologically or genetically HIF-1α-depleted mice, CNV was significantly suppressed with reduction of intraocular VEGF and/or ICAM-1 [23], [81]–[84]. Here, our results demonstrated that the HIF−1α activation induced by laser treatment was significantly suppressed by curcumin, which are compatible with previous results that curcumin or its derivate had the ability to down-regulated HIF−1α and VEGF expression in vascular endothelial cells and blocked angiogenesis *in vitro* [85]–[86]. Our data, at least in part, also suggest that the suppression of the expression of angiogenic and inflammatory molecules observed after laser injury is due to curcumin-induced inhibition of the NF-κB and HIF−1α pathway.

Collectively, the curcumin-mediated suppression of CNV formation observed in the present study is probably attributable to the inhibition of multiple inflammatory and angiogenic steps including the activation of NF-κB and HIF-1α, infiltration of macrophages and granulocytes, and up-regulation of inflammatory and angiogenic molecules. Therefore, we propose that curcumin may serve as a therapeutic approach to the treatment of CNV in AMD.
